# Rift Valley Fever Outbreaks in Mauritania and Related Environmental Conditions

**DOI:** 10.3390/ijerph110100903

**Published:** 2014-01-09

**Authors:** Cyril Caminade, Jacques A. Ndione, Mawlouth Diallo, Dave A. MacLeod, Ousmane Faye, Yamar Ba, Ibrahima Dia, Andrew P. Morse

**Affiliations:** 1 Institute of Infection and Global Health, University of Liverpool, 8 West Derby Street, Liverpool, L69 7BE, UK; 2 School of Environmental Sciences, University of Liverpool, Roxby Building, Liverpool, L69 7ZT, UK; E-Mail: A.P.Morse@liverpool.ac.uk; 3 Centre de Suivi Ecologique (CSE), Dakar, BP15532, Senegal; E-Mail: Jacques-andre.ndione@cse.sn; 4 Institut Pasteur de Dakar, Dakar, BP220, Senegal; E-Mails: diallo@pasteur.sn (M.D.); ofaye@pasteur.sn (O.F.); ba@pasteur.sn (Y.B.); dia@pasteur.sn (I.D.); 5 Department of Physics, University of Oxford, Robert Hooke Building, Oxford, OX1 3PU, UK; E-Mail: macleod@atm.ox.ac.uk

**Keywords:** Rift Valley Fever, Mauritania, Senegal, climate

## Abstract

Four large outbreaks of Rift Valley Fever (RVF) occurred in Mauritania in 1998, 2003, 2010 and 2012 which caused lots of animal and several human deaths. We investigated rainfall and vegetation conditions that might have impacted on RVF transmission over the affected regions. Our results corroborate that RVF transmission generally occurs during the months of September and October in Mauritania, similarly to Senegal. The four outbreaks were preceded by a rainless period lasting at least a week followed by heavy precipitation that took place during the second half of the rainy season. First human infections were generally reported three to five weeks later. By bridging the gap between meteorological forecasting centers and veterinary services, an early warning system might be developed in Senegal and Mauritania to warn decision makers and health services about the upcoming RVF risk.

## 1. Introduction

Rift valley fever (RVF) is a viral zoonosis that affects domestic animals and humans by causing an acute fever. This disease is caused by the RVF virus that belongs to the genus *Phlebovirus* in the family *Bunyaviridae*. The virus is transmitted to vertebrate hosts by the bite of infected mosquitoes, typically by the *Aedes* and *Culex* species. RVF mainly affects domestic animals (cattle, goats, sheep and camels, among others) and generally causes abortions in pregnant females and high mortality in young animals. Human acquire RVF through bites from infected mosquitoes or through exposures to infected animal material. Severe human infections are mainly caused by direct or indirect contact with viraemic animal blood or infected organs and foetuses (during butchering, slaughtering or veterinary procedures) and to less extent might also be caused by ingesting unpasteurized/uncooked milk from infected animals [1]. The human symptoms are characterized by the onset of high fever, headache, generalised weakness and liver abnormalities. In a small percentage of the infected human population the RVF virus can cause haemorrhagic fever, encephalitis and ocular disease and this can sometimes lead to death [2]. 

RVF is so called because the first outbreak was reported by Kenyan veterinary officers in the Rift Valley in 1915; the virus was then first isolated in 1931 [3]. Since then, numerous epidemic/epizootic outbreaks have been reported periodically in African countries and the Middle East (Yemen and Saudi Arabia) during the last thirty years [4,5,6,7,8]. In Mauritania and Senegal the first extensive RVF outbreak recorded occurred in 1987 and resulted in 220 human deaths [9]. Subsequently, several other outbreaks were recorded in 1993 [7], 1998 [10] and 2003 [11]. The RVF virus was isolated for the first time from *Culex* mosquitoes in Diawara, Senegal in 1998 [12]. This was carried out during an entomological survey undertaken in the area to assess the extent of the virus circulation in Senegal following the re-emergence of the RVF virus in south-eastern Mauritania. A total of 300 to 400 human cases and six deaths were reported during the 1998 outbreak that occurred in the province of Hodh El Gharbi in south-eastern Mauritania [10]. In 2003, twenty five confirmed human cases including sixteen with hemorrhagic signs and four deaths were reported in the Assaba, Trarza, Brakna, Tagant and Gorgol southern provinces of Mauritania [11]. In October and November 2010, a RVF outbreak occurred in three villages around the provinces of Atar and Inchiri in the northern part of Mauritania. According to the World Organisation for Animal Health (OIE) twenty one animal deaths were reported for these villages [13]; sixty three human cases including thirteen deaths were further reported at the end of December 2010 [14]. This was the first time RVF was reported in this region as the outbreaks generally occur in southern Mauritania [15]. Scientists and health and rural development ministers declared that the high rainfall conditions and warm temperatures experienced during the end of the rainy season might have favoured the development of RVF vectors over the area, leading to an increased disease risk [14]. In early September 2012, a first human case of RVF was reported in southern Mauritania, and by the end of October 2012, 34 human cases were further reported, including 17 deaths over the Assaba, Brakna, Hodh Chargui, Hodh El Gharbi, Tagant and Trarza southern provinces [16,17]. All human cases had history of contact with animals. Even if no animal clinical cases were reported by the OIE, abortions were observed in sheep and camels [16]. 

Climate variability has a large impact upon the incidence of vector-borne diseases: directly via the development rates and survival of both the pathogen and the vector, and indirectly through changes in the vegetation and the land-surface characteristics; this is particularly the case in Africa [18,19]. A significant relationship between RVF outbreaks, rainfall variability, land surface conditions and the El Niño Southern Oscillation has been highlighted over Kenya at the seasonal time scale [20]. However even if such correlation fits to east Africa, its application to west Africa remains so far unsatisfactory and is under investigation since preliminary studies have shown that the dynamics of RVF emergence seems to be more related to the distribution of rainfall during the monsoon season rather than to the total seasonal amount of precipitation. Indeed, studies carried out in northern Senegal showed that all RVF outbreaks observed over temporary pond areas in northern Senegal were related to similar rainfall conditions during the end of the monsoon season [21,22]. For all these events, a pause in precipitation followed by an intense rainfall event was observed during the late rainy season (August–September–October), just before the onset of RVF outbreaks. These meteorological events lead to the filling of temporary ponds, which might have resulted in the mass hatching of *Aedes* eggs infected by the RVF virus [21,22]. Because of vertical transmission of the RVF virus in *Aedes* eggs (the female mosquito infects its offspring) the virus chance to persist from one year to another is increased [21,22]. Recent findings further demonstrated that these meteorological events occur mainly over northern Senegal and southern Mauritania in West Africa and that the potential climatic risk of RVF is exacerbated by the presence of a large host reservoir (cattle, goats, sheep and to less extent camels) for these regions [23]. 

The main feature of the RVF outbreak over Mauritania in 2010 was its occurrence in an area where the surveillance system was almost non-existent. Therefore, local health services were confronted with a disease that never occurred in the Adrar region leading to severe socio-economic consequences. Given such impacts, the objectives of this study are to:

- Document the recent RVF outbreaks that occurred over Mauritania in 1998, 2003, 2010 and 2012 based on peer-reviewed publications and reports from governmental agencies.
- Study and quantify the environmental conditions that preceded RVF outbreaks in Mauritania.
- Discuss differences and similarities with east African RVF outbreaks and their link with environmental conditions, and provide recommendations for Mauritania.

## 2. Materials and Methods

### 2.1. RVF Mauritanian Outbreaks Details

Spatio-temporal details of major RVF outbreaks which occurred over Mauritania were obtained from different sources (peer-reviewed journals and FAO/OIE reports). These are summarized on Table 1. For 1998, only crude monthly information was available based on [10]. For the 2003 outbreak, detailed location of cases and timing of the outbreak were based on [11]. For the unusual 2010 outbreak, the reports of RVF cases in animals and people over Mauritania have been extracted from the World Animal health Information database (WAHID) interface [13]. For the 2012 outbreak we used recent data from [16] and [17]. 

### 2.2. Environmental Datasets

Daily rainfall is derived from the Tropical Rainfall Measuring Mission (TRMM) dataset (3b42 product, version 7, 0.25° spatial resolution). The TRMM product is a joint satellite project between NASA and JAXA designed to improve observations of precipitation over the tropics. The TRMM observations combine passive micro-wave, infrared and radar data from a constellation of satellite borne precipitation-related sensors and *in situ*-rain gauge products over 50ºN–50ºS [24]. Nicholson *et al.* (2003) showed that the TRMM merged rainfall product was in good agreement with rain gauge data over West Africa on monthly to seasonal time scales but they also highlighted biases at the daily time scale [25]. The new version (v7) has been improved and the TRMM data has been employed to model various health impacts such as malaria, cholera and meningitis [26]. 

Normalized Difference Vegetation Index (NDVI) data are based on smoothed NDVI outputs processed by the NOAA center for satellite application and research [27]. This data is based on NOAA AVHRR satellites; the NDVI data is available at 4 km^2^ and includes the study period (1998–2012). Animal densities (including sheep, goats and cattle) data have been derived from the FAO archive [28]. 

### 2.3. Method

**Figure 1:**
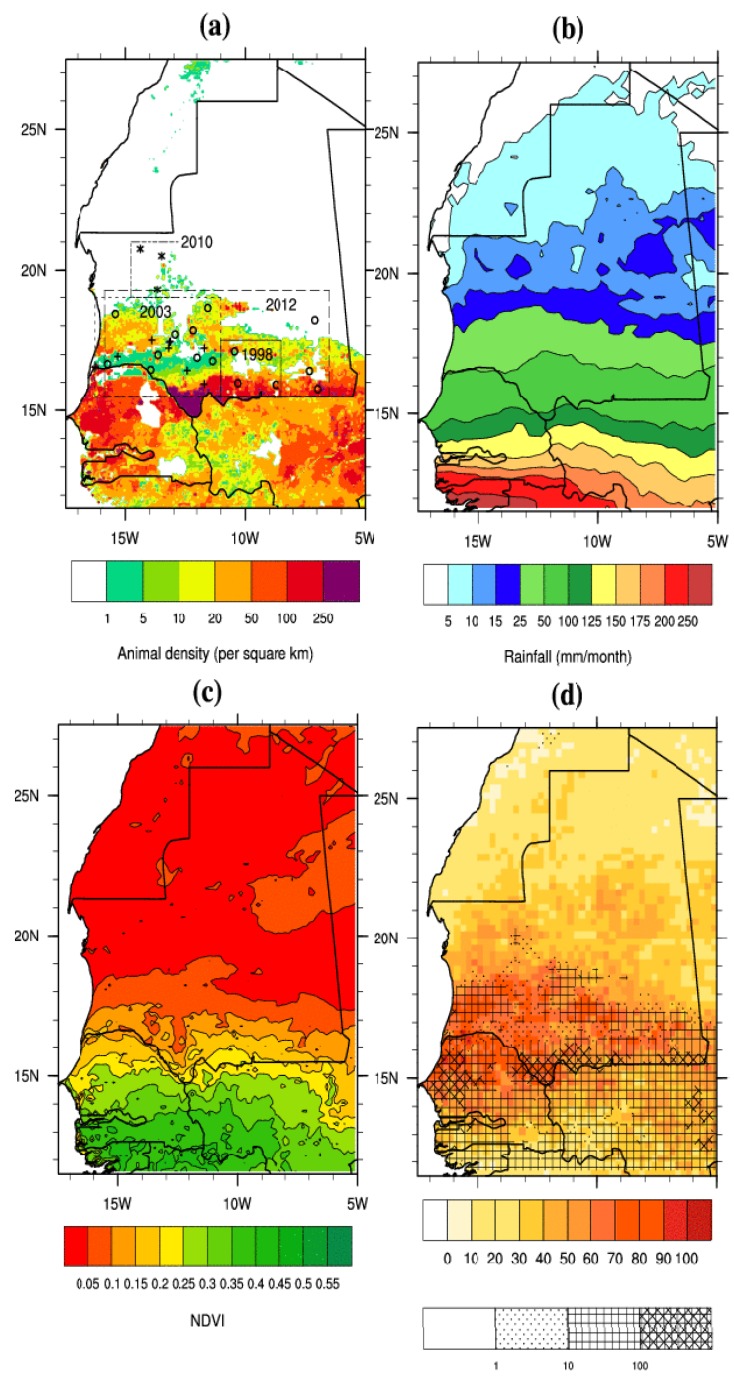
(**a**) Animal host density (sheep, goat and cattle) per km^2^ based on FAO dataset. The boxes depict the regions affected and the crosses, stars and circles depict the locations of reported infected cases in animals and humans for the 2003, 2010 and 2012 outbreaks respectively (see Table 1 for further details). (**b**) Rainfall climatology (July–August–September–October) based on TRMM satellite data (1998–2012). (**c**) Normalized Difference Vegetation Index (NDVI for August–September–October–November) based on the GVIX NOAA data (1998–2012). (**d**) RVF relative risk (%) based on TRMM rainfall data (1998–2012). The dotted, crossed and stippled areas depict animal host densities (sheep, goat and cattle) above 1, 10 and 100 per km^2^.

In order to investigate the relationship between the location and timing of RVF outbreaks with environmental parameters over Mauritania, we focused on rainfall and NDVI anomalies (departure from the mean e.g., the 1998–2010 climatology) as RVF outbreaks have generally been associated with heavy rainfall and increased NDVI conditions in eastern Africa which are related to warm phases of the El Niño Southern Oscillation [20]. We wanted to test those relationships for Mauritania. *Aedes vexans* mosquitos play a significant role in transmitting RVF to animals. Studies have shown that during the second half of the rainy season (August–September–October) rainless period of seven days (time needed for mosquito embryogenesis) followed by floods result in the hatching of very large numbers of new Aedes eggs, ultimately leading to increased RVF risk [21,22,29]. To investigate such a risk we tracked rainless period of seven days (seven consecutive days with no rainfall) followed by a rainfall event ≥10 mm occurring during the late rainy season e.g., August–September–October in the TRMM rainfall satellite estimates for the study period e.g., 1998–2012. If TRMM rainfall was below 1 mm for a given day/location we assumed no rainfall (e.g., we assumed it to be noise in the satellite data). The retained criteria is based on results highlighted by Mondet *et al.* [29] over Barkedji (northern Senegal) who showed that “Rainless period longer than seven days, the time needed for embryogenesis, followed by significant rainfall, will result in the hatching of very large number of eggs”. The 10 mm threshold was empirically derived from their results. RVF relative risk was then calculated by investigating the total number of those events over the period 1998–2012 and by rescaling those to range between 0% and 100% (Figure 1d).

## 3. Results

Figure 1a depicts the locations of the four main RVF outbreaks that occurred in 1993 over the Hodh El Gharbi region; in 2003 over a large area covering the Assaba, Trarza, Brakna, Tagant and Gorgol southern provinces of Mauritania (crosses); in 2010 over three villages in the Adrar arid northern region (stars); and in 2012 over southern Mauritania (circles). The animal host density is relatively small in northern Mauritania with respect to the large reservoir located near the southern boundary of the country where the largest outbreaks took place.

**Table 1:** RVF outbreak details for Mauritania (1998–2012).

Year	“Triggering Event” e.g., TE (Based on TRMM Rainfall)	1st Animal Infection	1st Human Infection	Source of Information	Time Difference between TE and 1st Human Infection
1998	7 September	September	September	Nabeth *et al.*, 2001 [10]	?
2003	1 October	November	Declared: 3 November Symptoms: 24 October	Faye *et al.*, 2007 [11]	3–4 weeks
2010	17 October	25 October	11 November	OIE, 2010 [13]	3 weeks
2012	11 August	?	16 September	FAO, 2012 [16] WHO, 2012 [17]	4–5 weeks

These density estimates do not include camels which were involved in RVF transmission in 2010 [14] and suspected to be involved in 2012 [16]. Mean rainfall over the area depicts a zonal pattern, with rainfall linearly increasing with latitude from the southern boundary of Senegal to southern Mauritania (Figure 1b). The Adrar where the 2010 RVF outbreak took place is arid, with rainfall ranging between 10 and 25 mm per month over this region. The rainfall distribution has a direct impact on the vegetation pattern with greener conditions shown over northern Senegal and southern Mauritania with respect to the north of Mauritania (Figure 1c). Relative RVF risk (see method for further details) is shown over Senegal and Mauritania on Figure 1d. This risk based on rainfall satellite estimates is high over northern Senegal and southern Mauritania where the animal density is large (stippled areas), and fits the spatial distribution of reported RVF cases in 1998, 2003 and 2012 (Figure 1a). This is consistent with results based on other rainfall datasets [23] and mapped endemicity estimates from the OIE and the Center for Disease Control (CDC). 

**Figure 2:**
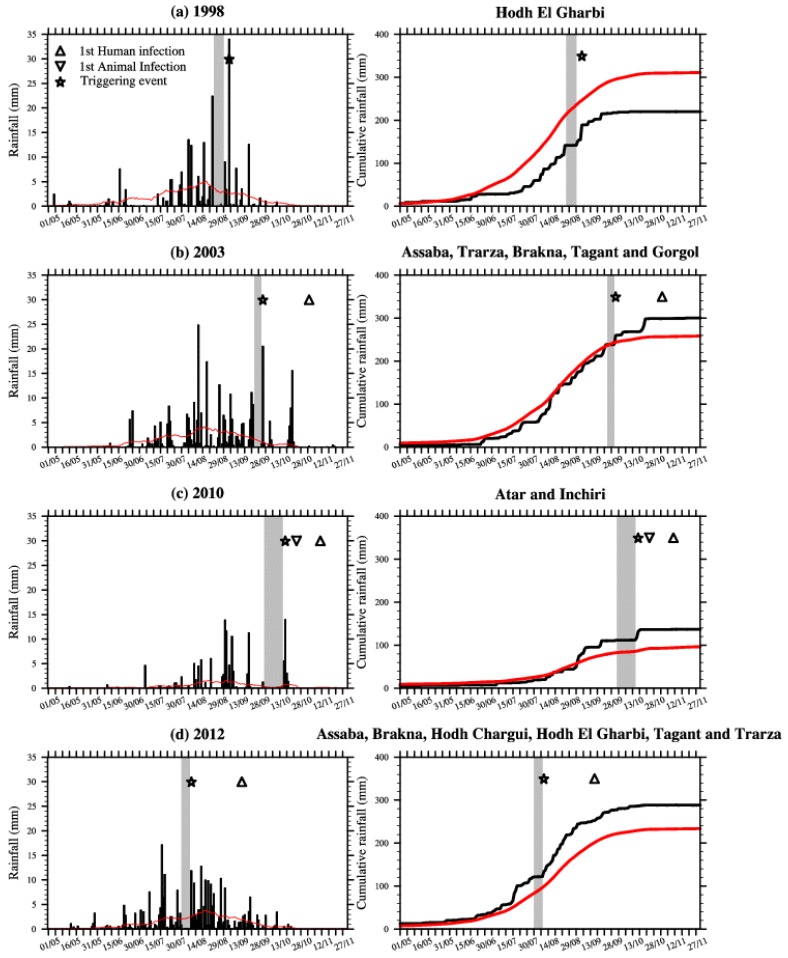
Rainfall indices (mm) averaged over (**a**) 15.5°N–17.5°N, 11°W–8.5°W in 1998, (**b**) 15.5°N–19°N, 16.25°W–11°W in 2003, (**c**) 19°N–21°N, 14.75°W–12.75°W in 2010 and (**d**) 15.5°N–19.25°N, 15.85°W–6.5°W in 2012. This is based on the TRMM rainfall dataset; see Figure 1 for a definition of the domains covering the RVF outbreak locations. Right: Cumulative rainfall (mm) for the same areas. The red line depicts the rainfall climatology (1998–2012) calculated for the respective areas. Dry spells are highlighted by the gray shading, large rainfall event are shown with stars and the first human and animal RVF reported cases are depicted by triangles.

The intra-seasonal rainfall variability over the affected regions in 1998, 2003, 2010 and 2012 is further investigated on Figure 2. The rainfall seasonal cycle over those regions exhibits a typical Sahelian pattern as the rainy season usually ranges from July to September with a peak centered in August. About 300 mm of rain are observed annually over the south-eastern and south-western regions whereas about 100 mm is observed over the northern arid region. 

In 1998 over the Hodh El Gharbi region, a dry spell occurred from 27 August to 3 September, with moderate rain occurring from 4 September to 6 September. This was followed by a large peak in rainfall (about 35 mm) on 7 September (Figure 2a). Rainfalls in September were heavy, but mean annual rainfall during that year was below the observed long-term rainfall climatology, so this was a relatively “dry year”. In September 1998, several patients were admitted to the Hospital of Aioun El Atrouss in the Hodh El Gharbi region with fever and haemorrhagic syndrome [10]. 

In 2003 over south-western Mauritania a rainless period was observed at the end of September (25–30), followed by a large convective event (more than 20 mm of rainfall) that traversed the area on 1 October (Figure 2b). This was followed by a long dry spell that lasted until 18 October. Then heavy rainfall was observed again from 20 October to 22 October. In October 2003, nine human cases of haemorrhagic fever were reported over the same region. The suspected case-patient 1 had onset of fever on 24 October and was admitted in Kiffa hospital on 3 November [11]. 

At the end of the 2010 rainy season over the Adrar region, unusual torrential rainfall also occurred. A rainless period starting on 25 September was followed by heavy rain conditions from 16–18 October (Figure 2c). The highest rains were recorded on 17 October (about 15 mm). The first animal cases were reported on 25 October in the village of Meddah in the province of Aoujeft; causing casualties in the sheep and goat population [13]. This was followed by two outbreaks that occurred on 10 and 11 November respectively in the villages of Tawaz and Akjoujt. In Tawaz, the RVF virus mainly affected camels causing high mortality, before being transmitted to humans, ultimately leading to thirteen human deaths [14]. The first human death related to RVF virus infection was further confirmed on 11 November. 

In 2012, a rainless period occurred from 4 August–10 August, followed by heavy precipitation on 11 August and regular rainfall until the end of the rainy season (Figure 2d). The first infected human case was reported earlier that season e.g., on 16 September, roughly four weeks after the large precipitation event occurred. 

These results for Mauritania confirm the observed relationship existing between rainless periods followed by large rainfall events and increased RVF risk at the end of the rainy season in northern Senegal [21,22,23]. The first infected human cases were generally reported about three to five weeks following the large precipitation event that was preceded by a rainless period (Table 1 and Figure 2).

**Figure 3:**
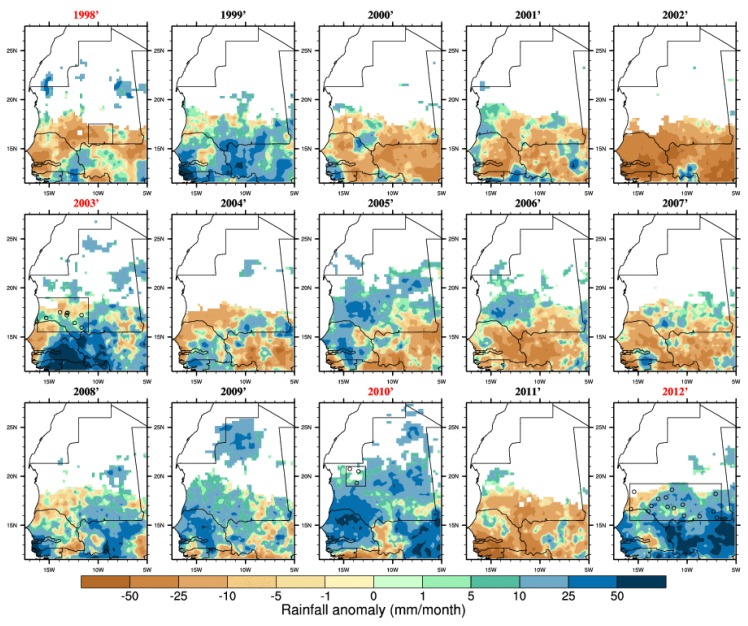
Rainfall anomalies (for the season June–July–August–September–October with respect to the 1998–2012 climatology) based on the TRMM satellite dataset. Outbreak years are highlighted in red and the locations of reported infections are shown by circles. Average rainfall below 10 mm for a given year has been masked (white area).

**Figure 4:**
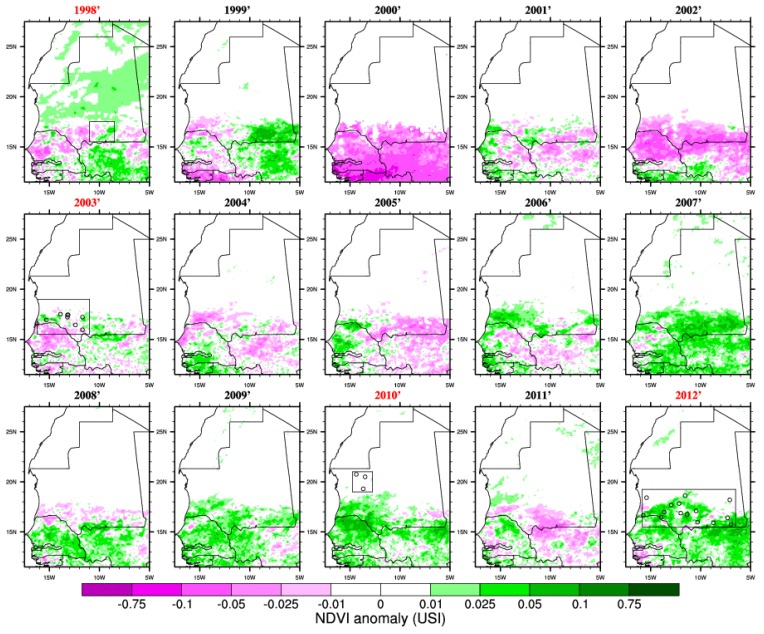
NDVI anomalies (for the season August–September–October–November with respect to the 1998–2012 climatology) based on the GVIX NOAA satellite dataset. Outbreak years are highlighted in red and the locations of reported infections are shown by circles.

**Figure 5:**
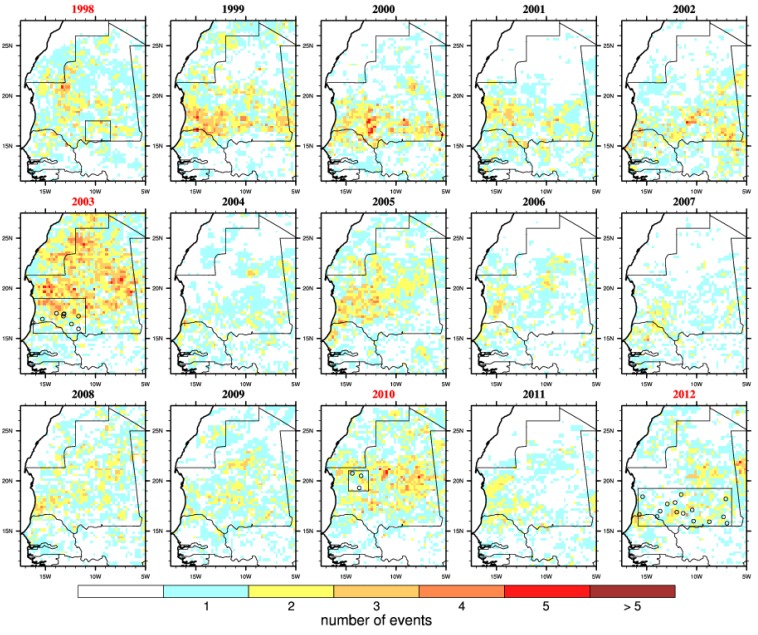
Number of embryogenesis risk events. This is defined by a dry spell (7 consecutive days with no rainfall) followed by a convective event (≥10 mm) occurring during the late rainy season (August–September–October). Outbreak years are highlighted in red and the locations of reported infections are shown by circles.

In order to test the RVF-climate relationship highlighted for eastern Africa over Mauritania, we investigated rainfall (Figure 3) and vegetation (Figure 4) distribution anomalies. We also mapped RVF risk for each separate year of the study period on Figure 5. The 2003, 2010 and 2012 events were associated with above average rainfall and increased vegetation conditions (Figure 3 and Figure 4). However, in 1998 the average rainfall was below the average so this was a relatively dry year (consistently with Figure 2). In 2005 and 2010 the rainy season was wetter than average especially over northern Mauritania where a RVF outbreak was reported in 2010. In general, the seasonal rainfall amount or the averaged vegetation conditions do not seem to be strictly related to RVF outbreak locations in Mauritania. As an example, south-western Mauritania experienced large precipitation and positive NDVI anomalies in 2009 but no outbreak was further reported. This significantly differs from the climate-disease relationship highlighted for eastern Africa.

A succession of rainless periods followed by heavy precipitation events (Figure 5) were observed in 1998, 2003, 2010 and 2012 near the locations of reported RVF cases. This is consistent with findings from Figure 2. Overall, the risk was the highest in 2003 over Mauritania when the largest RVF outbreak occurred. In 2010, those events occurred further north over the Adrar region. The risk distribution also matches well reported RVF cases distribution for 2012. However, those risk events also occurred over southern Mauritania from 1999 to 2002, over northern and western Mauritania in 2005 but no outbreaks were officially reported. In other words, this risk metric would also lead to a high proportion of false positive forecasts over the studied period.

The climate-disease relationship might be masked by the animal immunity to RVF amongst other socio-economic factors. Animals surviving RVF infection generally develop a lifelong immunity to RVF [30], and this is also true for humans [31]. The inter-epidemic RVF pause generally lasts 5 to 7 years in Senegal and this has been related to the livestock renewing period which is about 5 to 7 years for small ruminants [21]. This somehow fits with the time interval observed between the different reported outbreaks for Mauritania.

## 4. Discussion

Our findings corroborate that a rainless period lasting at least for a week, followed by large precipitation (at least above 10mm) at the end of rainy season (August–September–October) might have favored the risk in triggering RVF outbreaks in Mauritania in 1998, 2003, 2010 and 2012. This is consistent with results highlighted during four major RVF outbreaks that occurred in northern Senegal [21,22]. The risk in RVF transmission is also exacerbated by host promiscuity as the farmers generally water their cattle near the ponds during that period. This also can coincide with religious celebrations such as the Eid al-Adha and Eid al-Fitr which favor large concentration of ruminants and increase the number of potential contacts between humans and animals (critical factor). Our results confirm that the period for RVF emergence in arid and semi-arid West Africa is the late rainy season, generally during the months of September and October.

Rainfall intra-seasonal variability appears to be a more critical factor in triggering RVF risk in West Africa than the total seasonal amounts of precipitation or seasonal anomalies in NDVI (wet/dry or green/arid season) which are highly connected to RVF dynamics in eastern Africa [20]. As an example, an intense RVF transmission occurred in the Ferlo (northern Senegal) in 2003 that was a relatively dry year with respect to the long-term average [32]. 

Late rainfall events following a rainless period have been shown to impact on *Aedes vexans* mosquito population in Senegal [29]. The mechanisms that relate those meteorological events with RVF dynamics are now discussed. First, the temporary ponds are refilled almost synchronously by the heavy precipitation. The *Aedes* adult mosquitoes generally hatch four to five days after the rainy event, and a maximum period of ten days after a rain is generally utilized to conduct *Aedes* mosquito trapping in the field [29,33]. RVF risk is then enhanced due to the hatching of *Culex* mosquito which is already abundant during the end of the rainy season and the unusual hatching of *Aedes* mosquito during this time of year, as the eggs should have hatched during the following rainy season [29]. Indeed, the hatching of this *Aedes vexans* mosquito species is conditioned by a pause in precipitation followed by heavy rainfall conditions and generally occurs in phase with the rainy season; these eggs need a period of dormancy before being flooded so they can hatch. As the breeding habitats may contain vertically infected *Aedes* mosquito eggs (the virus being transmitted by the adult female to its offspring), the environment is favourable for the RVF virus due to both the possibility of its dispersion (unusual second peak of female *Aedes* mosquito occurring in September–October) and of its amplification (*Culex* hatching, with a peak in population occurring in September–October as well). The presence of both *Culex* and *Aedes* species during the late rainy season has been shown to be favoring RVF transmission over northern Senegal [32]. Once the hosts get in contact with the infected vectors, the virus incubation period is relatively short and ranges between 12 to 72 h in animals, and three to six days in humans. This time lag is consistent with the reported 2010 situation, as eight days separated abundant rainfall and the first reported RVF animal case in the first village (Table 1). The first human death was then reported on 11 November in the village of Tawaz but other socio-economic factors might have contributed to the spread of the RVF virus in humans. 

There are several caveats and limitations of this study that must be discussed. Firstly, the link existing between heavy precipitation following a dry spell and RVF risk might only be relevant for regions that experience a short rainy season, like regions located in the semi-arid Sahelian fringe (where most of the rains are concentrated from June to September). For these regions the pond/vector dynamic is strictly related to rainfall variability. In humid central and eastern Africa the land surface conditions (like soil moisture, vegetation, and landscape) might play a more important role. The availability of the pathogen is also a crucial point. As the Adrar region is a non-endemic part of Mauritania, there is no regular surveillance of RVF. This absence of data hampers our ability to discriminate how the mosquitoes became infected. A reasonable hypothesis is that the virus might have been introduced in the Adrar region in 2010 from viraemic animals transported by trucks from endemic southern Mauritania [14], but we cannot rigorously demonstrate the timing and the origin of the pathogen. The pathogen might then have spread through host promiscuity and due to the large pool of vectors available. Livestock movements appear to be a critical factor in driving RVF epidemics dynamics and they should be further investigated using other modeling approaches. Finally, livestock can also develop lifelong immunity to the RVF virus if they survive an outbreak [30] and this might mask this environment-disease relationship for the other studied years. This is consistent with results highlighted by Soti *et al.* [32], and with the number of false alarms that would have been forecasted with our method only based on environmental factors. As the livestock lifespan is about five to seven years for small ruminants, this might explain the lag observed between the 1998, 2003, 2010 and 2012 RVF epidemics in Mauritania. 

Human infections can be reduced by informing and educating farmers/herders about RVF. Preventive methods include precaution for animal milk and meat consumption, as well as avoiding direct contact with infected animal materials during slaughtering and veterinary practices. Concerning animal infections, sentinel herds in combination with disease early warning systems have been successfully utilized to anticipate RVF outbreaks in Kenya [34]. By bridging the gap between meteorological forecasting centers and veterinary services a RVF warning might be issued if a heavy rainfall event following a rainless period is forecasted during the second half of the rainy season over Senegal and Mauritania (using models and others means such as satellite images, radar, local expertise, *etc*.). But this would have to be complemented by information about the livestock susceptibility to RVF infections and this can vary considerably amongst different animals and breeds. The aggregated information can ultimately be broadcasted to the media, the policy makers and the health practitioners in order to warn the local population. However, the time lag between those events and the first reported human infections might be too short (a few weeks) to plan proper field intervention.

## 5. Conclusions

Four major Rift Valley Fever outbreaks occurred in Mauritania in September–October 1998, 2003, 2010 and 2012. Those outbreaks were preceded by heavy precipitation following a rainless period lasting a week that might have impacted on the *Aedes* vector population. The first human infection was generally reported three to five weeks following the late flooding. 
